# 2105. Early-onset and Late-onset BK Polyomavirus-associated Nephropathy: A 10-year Retrospective Analysis

**DOI:** 10.1093/ofid/ofac492.1727

**Published:** 2022-12-15

**Authors:** Vichitra Thiandech, Surasak Kantachuvesiri, Jackrapong Bruminhent

**Affiliations:** Faculty of Medicine Ramathibody Hospital, Mahidol University, Bangkok, Krung Thep, Thailand; Faculty of Medicine Ramathibody Hospital, Mahidol University, Bangkok, Krung Thep, Thailand; Faculty of Medicine Ramathibodi Hospital, Mahidol University, Bangkok, Krung Thep, Thailand

## Abstract

**Background:**

Polyomavirus-associated nephropathy (BKVAN) is one of the serious causes of allograft dysfunction and allograft loss in kidney transplant (KT) patients. Most of the patients were diagnosed with BKVAN within the first year after transplantation but some more cases of BKVAN occur later. A study focused on the differentiation between early and late-onset BKVAN is needed.

**Methods:**

We conducted a retrospective cohort study among KT recipients at Ramathibodi Hospital between January 2010 – December 2020. The incidence of early-onset (diagnosed within 1 year) and late-onset (diagnosed after 1 year) BKVAN, as well as composite kidney allograft outcomes, were compared.

**Results:**

From 1032 KT recipients, 645 (62.5%) were screened for BK viral infection. BKVAN was diagnosed in 83 (12.8%) patients. Of those, 46 (55.4%) and 37 (44.6%) were diagnosed with early-onset and late-onset BKVAN, respectively. Composite kidney allograft outcomes of GFR decline≥ 40% from baseline, graft loss or death were greater in early-onset BKVAN compared to late-onset BKVAN (7.5 vs 5.52 per 1000 person-months; hazard ratio 0.47; 95%CI, 0.23-0.95; P=0.037). In multivariate analysis, female gender and living-related kidney transplantation (LRKT) were independently associated with late-onset BKVAN compared to early-onset BKVAN. The more advanced the BKVAN diagnosis, the worse the allograft outcome occurs, even with optimizing immunosuppressive drugs and adjunctive therapy.

**Conclusion:**

BKVAN could occur later after one-year post-transplantation, although relatively better composite kidney outcomes were observed compared to early-onset BKVAN. Female individuals who underwent living-related KT should be considered for BK virus preemptive monitoring beyond one year to detect this late-onset BK virus infection.

**Disclosures:**

**All Authors**: No reported disclosures.

